# Utilization of a Clinical Trial Management System for the Whole Clinical Trial Process as an Integrated Database: System Development

**DOI:** 10.2196/jmir.9312

**Published:** 2018-04-24

**Authors:** Yu Rang Park, Young Jo Yoon, HaYeong Koo, Soyoung Yoo, Chang-Min Choi, Sung-Ho Beck, Tae Won Kim

**Affiliations:** ^1^ Clinical Research CenterAsan Institute of Life SciencesAsan Medical Center Seoul Republic Of Korea; ^2^ Department of Biomedical InformaticsAsan Medical Center Seoul Republic Of Korea; ^3^ Health Innovation Bigdata Center Seoul Republic Of Korea; ^4^ Office of Clinical Research InformationAsan Institute of Life SciencesAsan Medical Center Seoul Republic Of Korea; ^5^ Human Research Protection CenterAsan Institute of Life SciencesAsan Medical Center Seoul Republic Of Korea; ^6^ Department of Pulmonology and Critical MedicineAsan Medical CenterUniversity of Ulsan College of Medicine Seoul Republic Of Korea; ^7^ Department of OncologyAsan Medical CenterUniversity of Ulsan College of Medicine Seoul Republic Of Korea; ^8^ Clinical Trial CenterAsan Institute of Life SciencesAsan Medical Center Seoul Republic Of Korea

**Keywords:** clinical trial, information systems, academic medical center, information technology, privacy

## Abstract

**Background:**

Clinical trials pose potential risks in both communications and management due to the various stakeholders involved when performing clinical trials. The academic medical center has a responsibility and obligation to conduct and manage clinical trials while maintaining a sufficiently high level of quality, therefore it is necessary to build an information technology system to support standardized clinical trial processes and comply with relevant regulations.

**Objective:**

The objective of the study was to address the challenges identified while performing clinical trials at an academic medical center, Asan Medical Center (AMC) in Korea, by developing and utilizing a clinical trial management system (CTMS) that complies with standardized processes from multiple departments or units, controlled vocabularies, security, and privacy regulations.

**Methods:**

This study describes the methods, considerations, and recommendations for the development and utilization of the CTMS as a consolidated research database in an academic medical center. A task force was formed to define and standardize the clinical trial performance process at the site level. On the basis of the agreed standardized process, the CTMS was designed and developed as an all-in-one system complying with privacy and security regulations.

**Results:**

In this study, the processes and standard mapped vocabularies of a clinical trial were established at the academic medical center. On the basis of these processes and vocabularies, a CTMS was built which interfaces with the existing trial systems such as the electronic institutional review board health information system, enterprise resource planning, and the barcode system. To protect patient data, the CTMS implements data governance and access rules, and excludes 21 personal health identifiers according to the Health Insurance Portability and Accountability Act (HIPAA) privacy rule and Korean privacy laws. Since December 2014, the CTMS has been successfully implemented and used by 881 internal and external users for managing 11,645 studies and 146,943 subjects.

**Conclusions:**

The CTMS was introduced in the Asan Medical Center to manage the large amounts of data involved with clinical trial operations. Inter- and intraunit control of data and resources can be easily conducted through the CTMS system. To our knowledge, this is the first CTMS developed in-house at an academic medical center side which can enhance the efficiency of clinical trial management in compliance with privacy and security laws.

## Introduction

### Background

Clinical trials can pose potential risks and hurdles in communication between parties due to the many stakeholders involved, namely pharmaceutical companies, clinical research organizations (CROs), health authorities, ethical committees or institutional review boards (IRBs), courier vendors, and academic medical centers [1-4]. Given the wide scope and high volume of participants in clinical trials conducted nowadays, efficiency is a critical issue at site level during the trial [1,4]. These challenges arise due to the various authorities involved which gives rise to conflicting administrative processes, dysfunctional communications with the IRB, limitation of real-time data access for both investigators and authorities to consistently keep patients on track, limited personnel and/or infrastructural resources, noncompliance due to flaws in reporting, and omission of major events for awards and stipends. Furthermore, poor monetary management problems such as billing and checking problems can occur. These challenges can lead to subsequent controversy in future compliance [4-9].

To address the challenges outlined above, pharmaceutical companies and CROs have developed supportive tools such as clinical trial management systems (CTMSs) [2]. CTMSs enable the centralization of the clinical trial process, thereby reducing the number of procedural errors and enhancing communications among multiple stakeholders by providing timely metrics updates on a real-time basis [10]. Academic medical centers, however, have reported that proper utilization of commercial CTMSs poses certain operational hurdles [6,9-11].

### Objectives

As the academic medical center must manage the patient’s sensitive data compared with the pharmaceutical company, the security of the system should be considered in development. Moreover, academic medical centers have to make extra efforts to establish a system that is able to link pre-existing systems such as the health information system (HIS; subject management), electronic institutional review board (e-IRB; study approval), and enterprise resource planning (ERP) program (contract and budget of clinical trial) [12]. Therefore, the academic medical center can gain greater benefits from utilizing a centralized CTMS as its integrated research database, because copious volumes of medical data are often collected and saved in separately-operated systems. The overall objective of this study was to describe the methods, considerations, and recommendations for the development and utilization of CTMS as a consolidated research database in an academic medical center. We focused on the standardization of clinical trial processes and terminologies, enabling system interface among the legacy systems, and compliance with regulations of security and privacy of clinical data to better manage the whole process of clinical trials performed at academic medical centers.

## Methods

### Overview

This CTMS was launched in a tertiary hospital, Asan Medical Center (AMC). AMC is one of the largest academic medical centers in Asia, housing more than 2700 inpatient beds, and having around 10,000 patients visiting the outpatient clinics per day on average. Moreover, around 1100 clinical studies are initiated and conducted in the center every year, and it is one of the centers with the highest numbers of clinical trials in Korea. AMC has obtained and retained full accreditation from the Association for the Accreditation of Human Research Protection Program since 2013, and the AMC IRB has received accreditation from the Forum for Ethical Review Committees in the Asian and Western Pacific Region since 2006.

Since 1997, the AMC clinical trial center (CTC) has been designated as a specialized institution for conducting clinical trials. AMC CTC was designated as the Global Center of Excellence by the Ministry of Health and Welfare in 2012.

### Task Force Activities for System Development

The first step in the system development was to identify the tasks and longitudinal challenges that should be handled by the system, thereby establishing detailed system requirements. To identify and verify the system requirements from various stakeholders for developing a site-level CTMS, AMC organized a task force for 14 months from November 2013 to December 2014. The task force consisted of 17 stakeholders in clinical trial and research (including principal investigators, clinical research coordinator, clinical research associate, pharmacist, contract specialist, budget specialist information technology (IT) experts, relevant IRBs, and information protection subcommittee). All authors of this paper participated in this task force. Following 6 topics are discussed in this task force: (1) define clinical trial processes at the site- and unit-specific level, (2) define data governance and access rules for each member and member’s unit, (3) define and match controlled vocabulary and annotation rules to represent clinical trial data, (4) define system requirements for each department, (5) define an interfacing strategy between CTMS and legacy systems (e-IRB, ERP, and HIS), and (6) define data protection strategies based on international and domestic law. For this purpose, 56 stakeholders were interviewed 25 times, and the resulting data were used by the CTMS task force for developing site-level CTMS. On the basis of these results from CTMS task force, we define a strategy, a process, and guidelines for building and managing site-level CTMS.

### System Development

The basic architecture for building CTMS was defined as follows, which involved all members of the aforementioned CTMS task force. The first step to building CTMS was defining the trial processes at the site level. The next step was to define and extract vocabulary from actual clinical trial data. The vocabulary set was matched with the standard terminology, namely the Clinical Data Interchange Standards Consortium (CDISC)-controlled vocabulary version P29 [13]. We then identified the tasks of all departments involved in the clinical trial and collected the system requirements through the analysis of necessary functions and data for each task (needed for computerization and collaboration, characteristics of generated data, access to data, etc). Table 1 shows the categories and features for management of overall clinical trials at the site level.

Finally, we designed the system architecture for developing CTMS at the site level, which includes modeling the database scheme, specific user interface (UI), and user experience (UX) design. In all the above system development processes, we referenced official guidelines from the US Food and Drug Administration (FDA) and the Australian Therapeutic Goods Administration (TGA). The US FDA has outlined a guidance document addressing computerized systems used in clinical investigations, otherwise known as 21 Part 11 [14] and the Australian TGA has developed a set of notes on Good Clinical Practice (GCP) [15]. They are used to protect research subjects, manage consent, and comply with reporting obligations to regulatory agencies such as IRB, US FDA, and Korean Ministry of Food and Drug Safety. Each function of the CTMS is intended to ensure that key stakeholders in clinical trials effectively comply with GCP and FDA 21 Part 11, the regulation for managing electronic clinical trial data. For example, we implemented the single sign-on function for authority check, leaving a log of all events occurring in the CTMS and managing the training log of the participants. To protect personal information, the Health Insurance Portability and Accountability Act (HIPAA) and Korean Privacy Act have been followed when designing and building the system.

A total of 12 months was spent on system development. The first 3 months were spent for collecting unit and user requirements, 2 months were spent on designing systems such as the database scheme, UI, UX, and terminology, 5 months were spent on developing, and the remaining 2 months were spent on system deployments. The evaluation of this system was carried out by monitoring the change of clinical test management flow in the schedule management for subjects according to CTMS introduction. AMC CTMS was built in the IBM Advanced Interactive eXecutive 6.1 server using JAVA 6, Oracle 11g, Apache 2.2.27, and Weblogic 10.3.6.0. CTMS can be accessed via all major internet browsers such as Chrome, Safari, Internet Explorer, Edge, and Firefox. Communication between server and system was secured with a Hypertext Transfer Protocol Secure in combination with a Secure Sockets Layer or Transport Layer Security protocol.

**Table 1:** The categories and features for management of overall clinical trials at the site level. The clinical trial management system (CTMS) features are divided into 3 types: inputting data, receiving an interface through a different system, and automatically calculating.

Category	Features	Description
Study management	Study list^a^	The study listed in the CTMS^b^ is an IRB^c^ approved study. IRB and CTMS are shared daily with the system interface. Additional study may be registered by the user
	Status history^d^	Ability to show a record of all events (additions, modifications, deletions) taking place in the study
	Milestone management^d^	Ability to manage timeline of study (eg, Regulatory Complete)
	Contract management^d^	Ability to manage clinical trial contract information (contract timeline, stakeholders, negotiations, etc)
	Budget management^d^	Management functions for budgeting and execution of research funds
	Document management^d^	Ability to manage all documents generated during clinical trials (separated by department)
Site management	Site management^d^	Ability to manage information about another site when carrying out multisite research by ARO^e^
	Communication and contact information management^d^	Ability to manage communication with a site or other organization by ARO
	Investigational product^d^	Ability to manage information about interventional product, vendor, sponsor by ARO
Subject management	Subject management^f^	Ability to manage subject information automatically interface with HIS^g^
	Subject scheduling calendar^f^	Ability to manage the subject's schedule is automatically displayed in calendar form through the patient management function
	Quality control management^d^	Ability to manage the quality control of subject includes informed consent, adverse event, protocol deviation, and inclusion or exclusion criteria managing for each subject
	Healthy volunteer announcement list^d^	Ability to manage the registration of healthy volunteer recruitment announcement and healthy volunteer pool management
Clinical monitoring	IRB regulatory list^d^	IRB approval and related data management ability for each site in case of multisite research
	MFDS^h^ regulatory list^d^	MFDS approval and related data management ability
	Site visit list^d^	Ability to manage information about site visit status and results
	Protocol deviation list^d^	Ability to manage information related to site-specific protocol deviation
	SAE^d,i^ list	Ability to manage information related to site-specific SAE
External request management	Feasibility request management^d^	Ability to manage feasibility request from sponsors
	PRIMS^d,j^	Provides clinical trial advisor request management from external pharmaceutical companies
	Clinical trial management of medical devices^d^	Provides clinical trial management functions related to medical devices coming from external organizations
Resource management	Medical equipment management^a^	Ability to manage medical device calibration information
	Drug management^d^	Ability to manage the clinical drug import and export
	Biomaterial management^a^	Ability to manage the biomaterial obtained during clinical trials
	Monitoring room management^d^	Ability to manage spaces for monitoring by external clinical research associate (CRA)
	Investigator profile^d^	Ability to manage investigator profile of our organization
Education management	Education management^d^	Ability to manage training for clinical trial worker in inside and outside organization
	SOP^d,k^ Education	Supports SOP management, training, and automatic notification by the unit
Unit management	Unit member management^d^	Support for unit member management, CTMS page access right and position management
	Member education management^d^	Demonstrate the data generated by the education manager for the education management of unit members
	To-do list management^e^	Provides management functions for tasks to be performed by each user. The task is automatically generated by CTMS such as request confirmation to obtain clinical patient consent form

^a^The interface.

^b^CTMS: Clinical Trial Management System.

^c^IRB: Institutional Review Board.

^d^Inputted directly by the user.

^e^ARO: academic research organization.

^f^Automatic calculation.

^g^HIS: Health Information System.

^h^MFDS: Ministry of Food and Drug Safety.

^i^SAE: Serious Adverse Event.

^j^PRIMS: Preclinical and eaRly ClInical Support program.

^k^SOP: Standard Operating Procedure.

## Results

We successfully developed and introduced the CTMS to AMC to manage the large amount of data involved in clinical trial operations. This system was designed for inter- and intraunit control of data and resources to improve efficiency in clinical trial management.

### Site Level Clinical Trial Process

**Figure 1:**
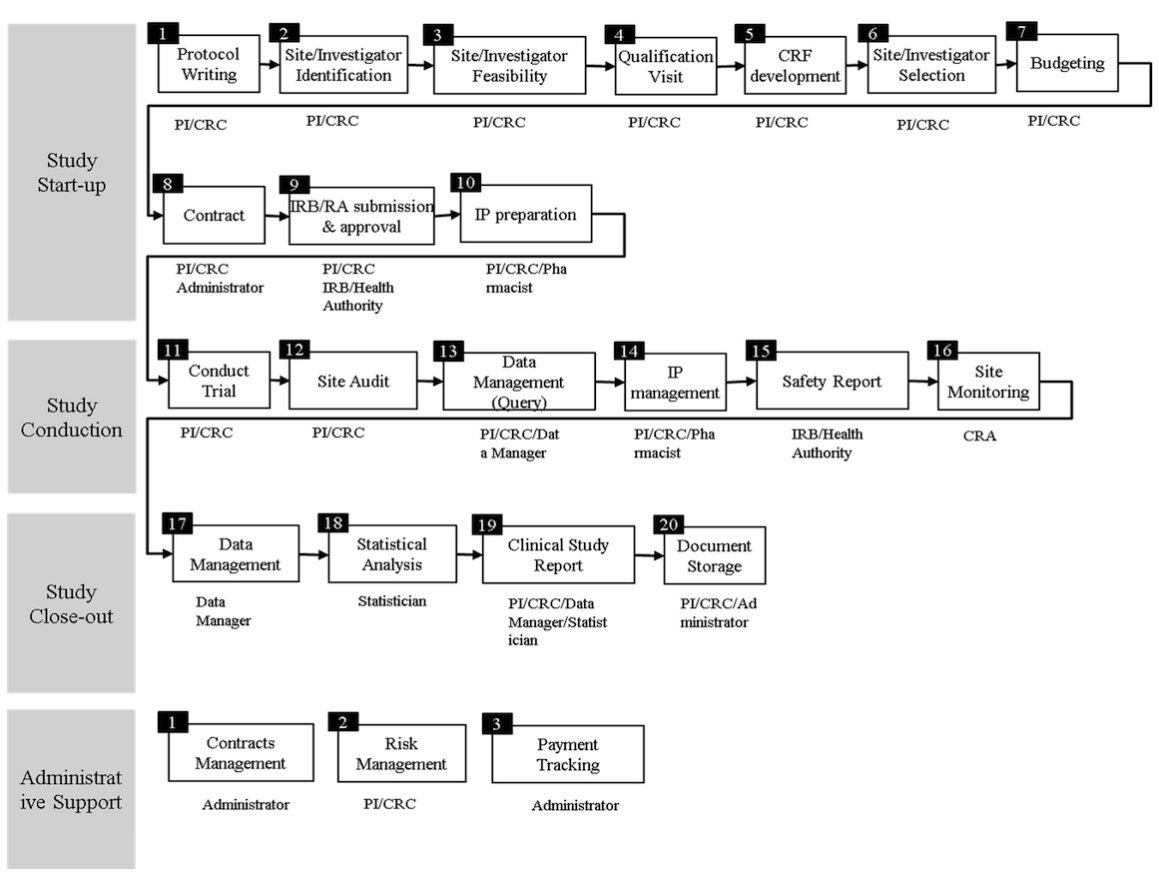
A designed clinical trial process for the Clinical Trial Management System. CRA: clinical research associate, CRC: clinical research coordinator, CRF: case report form, IP: intellectual property, IRB: institutional review board, PI: principal investigator.

The general processes of a clinical trial at the site level were established and grouped into 4 important milestones (namely study start-up, study conduction, study close out, and administrative support) and then further categorized into 23 internal processes (Figure 1). We defined 4 groups of milestones to include all start-up activities from protocol writing to intellectual property preparation, clinical trial conduction step including site monitoring, trial close-out step to cover document storage process, and, finally, administrative support that covers contract management and payment tracking support for the overall clinical trial process.

### Controlled Vocabulary for Clinical Trial

Ensuring the uniformity of terms is crucial when creating interdepartmental collaboration systems. In this study, a set of related terms for each process were defined after examining each department’s clinical trial processes to ensure the consistency. We extracted 1354 terms at the raw level by dividing them into 23 individual clinical trial processes (defined in the Results section), and into 7 clinical trial tasks, namely study management, resource management, healthy volunteer, report management, education management, user and organization management, and administrative management. A team consisting of 1 medical doctor, 2 clinical research coordinators (CRCs), 2 medical records technicians, and 1 IT professional mapped the raw level terminology used in the local terms used at each site and produced 643 mapped terms. The defined 643 terms were created in 2 languages—Korean and English—and the terms were used throughout the construction of the CTMS system. We also performed a mapping with the CDISC Controlled Terminology, which is a representative term for clinical trials, to verify the representativeness of the term. As a result, almost two-thirds of the terms (421/643, 65.4%) were verified to have been mapped correctly.

### System Architecture

**Figure 2:**
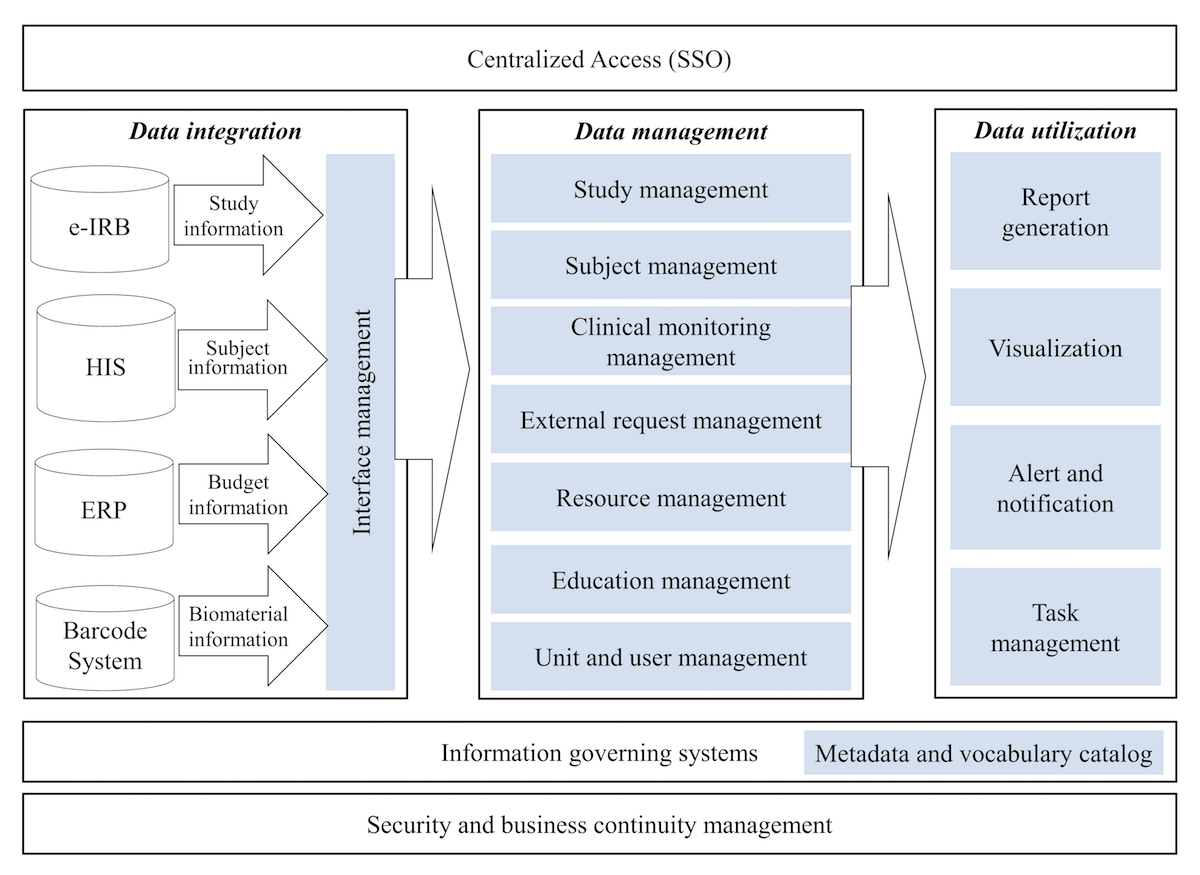
System architecture for Clinical Trial Management System. e-IRB: electronic institutional review board, ERP: enterprise resource planning, HIS: health information system, SSO: Single Sign-On.

The AMC CTMS is an all-in-one system containing all functions needed for operating site-level clinical trials and is a Web-based single sign-on system. As shown in Figure 2, the AMC CTMS is mainly composed of 3-layered domains, namely data integration, management, and utilization. First, the data integration layer of the system includes functions that interface data between the CTMS and legacy systems such as e-IRB, HIS, ERP, and barcode system in the hospital. Data for each existing system are unified and transmitted to the CTMS according to the system-specific interfacing cycle. Depending on the characteristics of the legacy system, the interface methods with the CTMS are different: (1) large scale systems such as the HIS and ERP systems were made through a separate interface server, (2) e-IRB as a Web-based system is linked to a Web service via the Application Programming Interface, and (3) the barcode system as a standalone system is linked through a database to database link. The data management layer of the system includes 7 management applications for basic study management and for managing operational data for clinical trials at the site level. Finally, the data utilization layer of the system is composed of the following 4 applications—report generation, visualization, alert and notification, and task management. The 4 applications in this domain are based on operating data in the CTMS to support clinical trial operations, such as notifications of subject visits to the hospital.

### Data Governance and Access Rules

As AMC CTMS was designed to be used by various people both inside and outside the clinical trial site, the CTMS task force created rigorous data governance and access rules to protect both the system and the data. In terms of data access authority, the basic unit of the CTMS is the study, and the access right of the study is defined according to the IRB approval. Page access rights are classified according to departmental business characteristics. Every page is clearly distinguished between the subjects of management and use. For example, the Biomaterial Management page is the subject of management of the clinical pharmacokinetics laboratory, but the subject of use is all researchers and CTC members. External user registration is the responsibility of the system administrator, and only for companies previously registered with IRB. Furthermore, access rights are also granted in a limited manner.

### Privacy and Security

To protect sensitive patient information, 21 personal health identifiers (PHIs) were excluded from the CTMS to comply with international and domestic law. Table 2 shows the 21 PHIs adopted by AMC for developing a de-identified Clinical Data Warehouse. As a result, a CTMS user can identify the subject based on the subject’s number assigned for each clinical trial and their date of birth. Systematically, the patient’s registration number in the hospital is encrypted and stored in the CTMS database, which is linked to the HIS data for automatic data transferring. A more detailed description of the 21 PHIs determined by AMC has been described previously [16,17].

**Table 2:** The 21 Personal Health identifiers adopted by the Asan Medical Center from Table 1 in Shin et al reference number [17] (adapted with permission).

Number	Identifier	Remarks	Reference
1	Name	Excludes physician’s name, includes information regarding friends and relatives.	HIPAA^a^ safe harbor; HIPAA LDS^b^
2	Address	Smaller than the submunicipal level divisions (Dong, -Eup, and -Myeon).	HIPAA safe harbor; HIPAA LDS
3	Phone number	Includes mobile phone and fax numbers.	HIPAA safe harbor; HIPAA LDS
4	Email address		HIPAA safe harbor; HIPAA LDS
5	Resident registration number		Korean Personal Information Protection Act
6	Foreigner registration number		Korean Personal Information Protection Act
7	Passport number		Korean Personal Information Protection Act
8	Health insurance policy number		HIPAA safe harbor; HIPAA LDS
9	Bank account number		HIPAA safe harbor; HIPAA LDS
10	Credit card number		HIPAA safe harbor
11	Certiﬁcate or license number	Driver’s license	Korean Personal Information Protection Act; HIPAA safe harbor; HIPAA LDS
12	Vehicle license plate number		HIPAA safe harbor; HIPAA LDS
13	Patient identifier	Medical record numbers	HIPAA safe harbor
14	Hospital membership ID	Hospital homepage, referral system	Korean Act on Promotion of Information and Communication Network Utilization and Information Protection
15	Hospital employee number		HIPAA safe harbor
16	IP address		HIPAA safe harbor; HIPAA LDS
17	URL		HIPAA safe harbor; HIPAA LDS
18	Biometric identiﬁer	Fingerprints, retina, vein, voice prints, and personally identiﬁable genetic information	HIPAA safe harbor; HIPAA LDS
19	Full-face photographic images and any comparable images		HIPAA safe harbor; HIPAA LDS
20	Birth date (allowing year and month)	For example, July 1960 can be used, but July 4, 1960, should be used as July 1960	HIPAA safe harbor
21	Other unique identifying numbers	Pathology numbers	HIPAA safe harbor

^a^HIPAA: Health Insurance Portability and Accountability Act.

^b^LDS: Limited Data Set.

The CTMS system goes through a pre-inspection in accordance with the privacy principle guidelines. The privacy principle guidelines are broken down into 64 items, and this is performed on a nonregular basis by the Ministry of Government Administration and Home Affairs of South Korea. A simulation hacking test was conducted based on 3 steps, namely information gathering, vulnerability categorization, and penetration phase, by configuring the major vulnerabilities of the system from the 2013 Open Web Application Security Project’s 10 weak points, and the National Intelligence Service’s 8 weak points.

### Evaluation

The evaluation of the CTMS was performed by examining scenarios related to schedule management for subjects in clinical trials. Before the introduction of the AMC CTMS, clinical trial stakeholders proceeded with their own research in isolation. For example, to manage subject schedules, the CRC used a personalized tool such as Google Calendar, and the record was not reused. In the CTMS, each research study is assigned a specific study design (Multimedia Appendix 1). If the user is registered as a participating researcher upon approval of the IRB study, the assignment can also be accessed in the CTMS.

**Figure 3:**
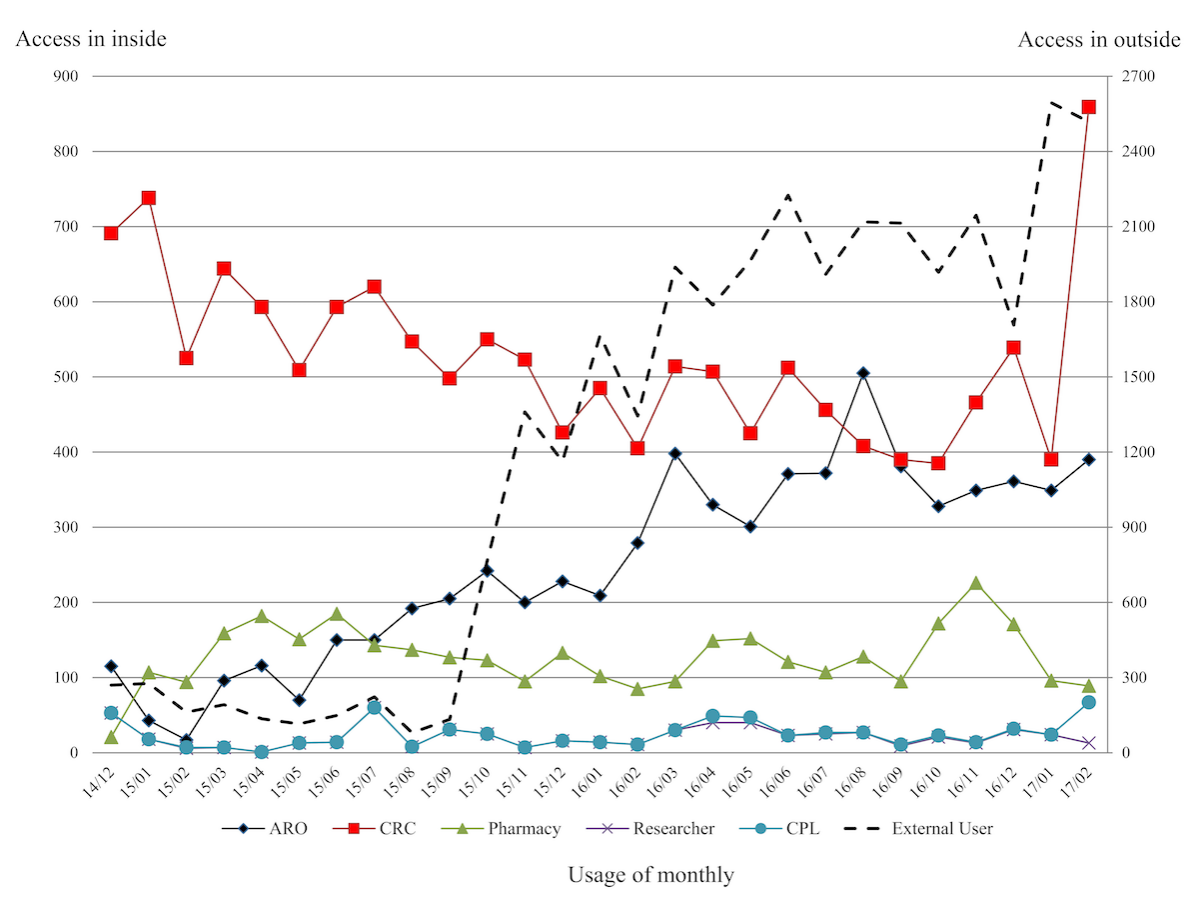
Monthly access trend of Clinical Trial Management System by user’s occupation. ARO: academic research organization, CRC: clinical research coordinator, CPL: clinical pharmacokinetics laboratory.

When a subject is recruited, the patient’s schedule is shown in a calendar format in the study design data, which is essentially controlled by the CRC, although the study participant has the right to retrieve these data (Multimedia Appendix 2). If there is a schedule for export of clinical trial drug, these data will be forwarded to the pharmacy in advance and a record of the amount of exported drug is stored in the CTMS (Multimedia Appendix 3). As a result, within the CTMS, all stakeholders can use and view the same data with varying privileges in terms of patient schedule management.

### Deployments

Since December 2014, the CTMS has been successfully implemented at AMC. To utilize the data before the introduction of the system, data loading work was carried out 2 months before the system opened (approximately 8000 studies and 100,000 patients’ information with related data). As a result, by March 31 2017, a total of 11,645 studies have been managed by 881 users in the AMC CTMS. Moreover, a total of 146,943 subjects have been enrolled in the AMC CTMS, including those who were managed by the pre-existing HIS. There are 1316 external users, including CRA from CROs and trainees. Figure 3 shows the monthly access trend of the CTMS according to user’s department. We observed distinct differences in the CTMS access rates according to the occupation of the user in the hospital. For example, the ARO showed a steadily increasing pattern (*R*^2^=.81), whereas the CRC decreased, but the access rate suddenly increased due to the implementation of additional functions for CRC. The utilization rate for external users showed a dramatically increasing overall pattern (*R*^2^=.85).

## Discussion

### Principal Findings

Clinical trial data management and data quality control pose challenging tasks in many organizations participating in clinical trials [1,3], especially in academic medical centers where the environment is optimized for patient care and not clinical trials [4,18]. In pharmaceutical companies and CROs, CTMS are generally developed to match the characteristics of the tasks performed by the institution. Many academic medical centers are not only concerned with conducting clinical trials, but also have an obligation to collect and retain data related for the whole clinical trial period while maintaining a sufficiently high level of data quality [1,4,12]. In recent years, the obligation to monitor the ARO function has been added for better management of investigator-initiated trials.

Although the introduction of commercial or open-source systems can be considered at academic medical centers, many existing studies have noted limitations in using these systems at this type of medical center [6,9-11,19,20]. The biggest problem with commercial or open source CTMSs is that it is difficult to customize a fixed workflow [10,19,20]. These problems, therefore, make it difficult for such a CTMS to manage a variety of clinical trials, which is often necessary at academic medical centers, or clinical trials involving many stakeholders simultaneously. To address these burdens of covering a wide scope of work, we designed and constructed a site-level CTMS according to the following four important aspects. First, we established an agreement among the stakeholders involved in clinical trials. For this, we formed a CTMS task force with various stakeholders participating in clinical trials for 14 months, a period during which we covered all phases of CTMS design, implementation, and deployment. During the 14 months of the task force operation, interviews on various departments were conducted to organize and integrate the requirements for designing CTMS.

Second, a standardized clinical trial process was established and the unified terminology at our academic medical center was listed. As mentioned in the Methods section, different types of clinical trials were performed at the site with different study phases and therapeutic areas. There were also some cases in which only a part of the clinical trial was conducted according to the contract. To arrange these heterogeneous tasks in clinical trials, institutional standardization of clinical trial processes and terminology was defined.

Third, we built strong interfaces between the CTMS and legacy clinical trial support systems such as HIS, e-IRB, ERP, and biomaterial management system. On the system side, the most important aspect of site-level CTMS is its seamless operation with the legacy systems. We conducted a thorough review with the hospital’s IT development team to determine which data would be linked at any given time and whether there were any potential privacy or security issues with the associated data.

Lastly, the CTMS was designed in accordance with domestic and international security and privacy protection regulations for compliance perspectives. As an operating system that deals with sensitive patient information, the system was designed based on various laws and regulations in Korea and abroad, and it has been verified as secure by the hospital’s IT development team.

### Strengths and Limitations

The primary advantage of utilizing a CTMS at an academic medical center is that it makes it easier to track clinical trial progression and capture the whole process of the clinical trial with minimal human efforts using a standardized IT system. As clinical trials are performed by multiple stakeholders, it is crucial to have all stakeholders consistently updated of any progress and to be on the same page with every decision made. The conventional way of reporting clinical trial progress to stakeholders often resulted in miscommunications as stakeholders delivered messages and updates via their preferred way of communication. This could range from phone calls to emails, or from fax to hard copies of letters. This mode of communication lends itself to the omission of important stakeholders from the loop of communication, and various stakeholders could often miss data if important information is not communicated in a timely manner. To address these issues, many pharmaceutical companies have actively tried to build standardized communication tools by recruiting support from their IT development teams. In this regard, CTMSs have proven to be an effective tool for managing clinical trials. Principal investigators who often simultaneously conduct multiple clinical trials received reports on the progress of all ongoing clinical trials on a real-time basis and were able to effectively manage their trials that involved dozens of responding staffs, such as sub-investigators, CRCs, CRAs, pharmacists, and administrative staffs, as they make use of the CTMS as a single consolidated database. As principal investigators could follow the study progress on a real-time basis, it became easier to promptly address any issues identified during the course of clinical trials.

Moreover, the CTMS significantly assisted for the leadership in academic medical centers because the CTMS tracks the performance of multi-stakeholders and extracts data that reflects utilization of resources, holding issues, bottleneck steps, and so on. Instead of receiving dispersed reports from each individual function, it is now possible for the leadership of academic medical centers to track the performance of each study at any time, to resolve any issues, and to make informed management decisions.

One of the limitations of this study is that the feasibility of the CTMS development is only confirmed by a single academic medical center. Although the volume of clinical trials is large and the CTMS covers different types of clinical trials that reflect different standard operating procedures of various pharmaceutical companies, its data were extracted only from a single-frame platform. Thus, data from academic medical centers of varying institution size and heterogeneous IT infrastructures need to be investigated in a future study. Recently, the FDA has created a Clinical Trial Transformation Initiative to improve the quality and efficiency of clinical trials [21]. This initiative is underway for safety reporting and data monitoring committee project to improve clinical trial safety. Further research is needed to improve the CTMS to flexibly reflect these regulatory policy changes.

### Conclusions

As the academic medical center has a responsibility and obligation to conduct and manage clinical trials while maintaining a sufficiently high level of quality, it is necessary to build an IT system for supporting standardized clinical trial processes and to comply with relevant regulations. In this paper, we propose the methods, considerations, and recommendations for development and utilization of a CTMS as a consolidated research database in an academic medical center. We have outlined the benefits of adopting a CTMS based on specific scenarios, but further studies on efficiency and accuracy based on data are needed.

## Appendix

Multimedia Appendix 1 Screenshot of study design on the Asan Medical Center (AMC) Clinical Trial Management System (CTMS). Sensitive items in terms of study information and privacy protection were masked. The original webpage shows only the Korean menu names. They were translated into English for international users.

Multimedia Appendix 2 Screenshot of calendar of subject scheduling in the Asan Medical Center (AMC) Clinical Trial Management System (CTMS). Sensitive items in terms of study information and privacy protection were masked. The original webpage shows only the Korean menu names. They were translated into English for international users.

Multimedia Appendix 3 Screenshot of drug management in Asan Medical Center (AMC) Clinical Trial Management System (CTMS). Sensitive items in terms of study information and privacy protection were masked. The original webpage shows only the Korean menu names. They were translated into English for international users.

## Abbreviations

ARO: academic research organization
AMC: Asan Medical Center
CDISC: Clinical Data Interchange Standards Consortium
CRA: clinical research associate
CRC: clinical research coordinator
CRO: clinical research organization
CTC: clinical trial center
CTMS: clinical trial management system
e-IRB: electronic institutional review board
ERP: enterprise resource planning
FDA: Food and Drug Administration
HIPAA: Health Insurance Portability and Accountability Act
HIS: health information system
LDS: Limited Data Set
TGA: Therapeutic Goods Administration
UI: user interface
UX: user experience
