# Supplementary figures and images for: Utilization of a Clinical Trial Management System for the Whole Clinical Trial Process as an Integrated Database: System Development
Source: J Med Internet Res. 2018 Apr 24;20(4):e103. doi: 10.2196/jmir.9312 (PMC5941091; doi:10.2196/jmir.9312)

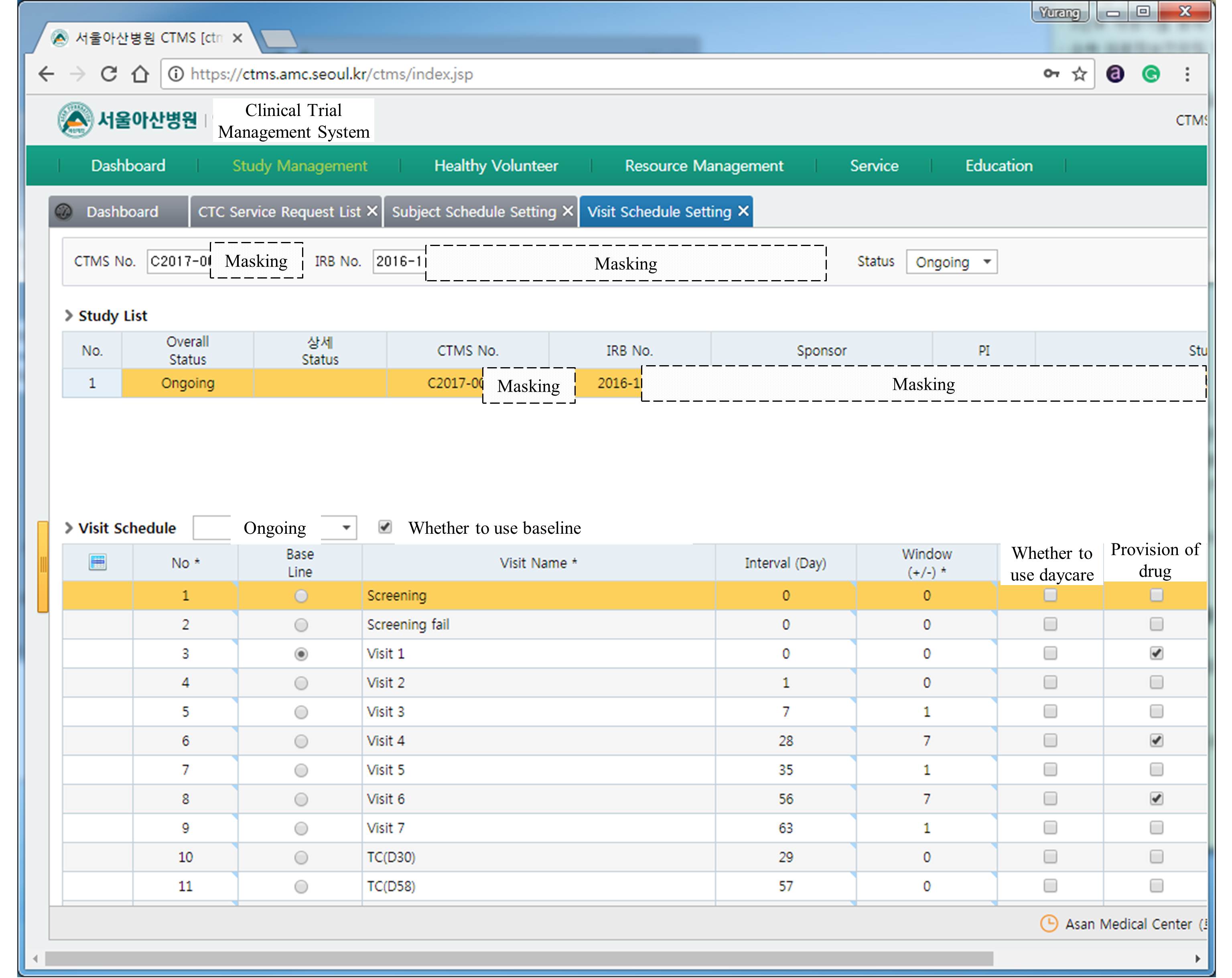

Supplement: Multimedia Appendix 1 [file jmir_v20i4e103_app1.jpg]

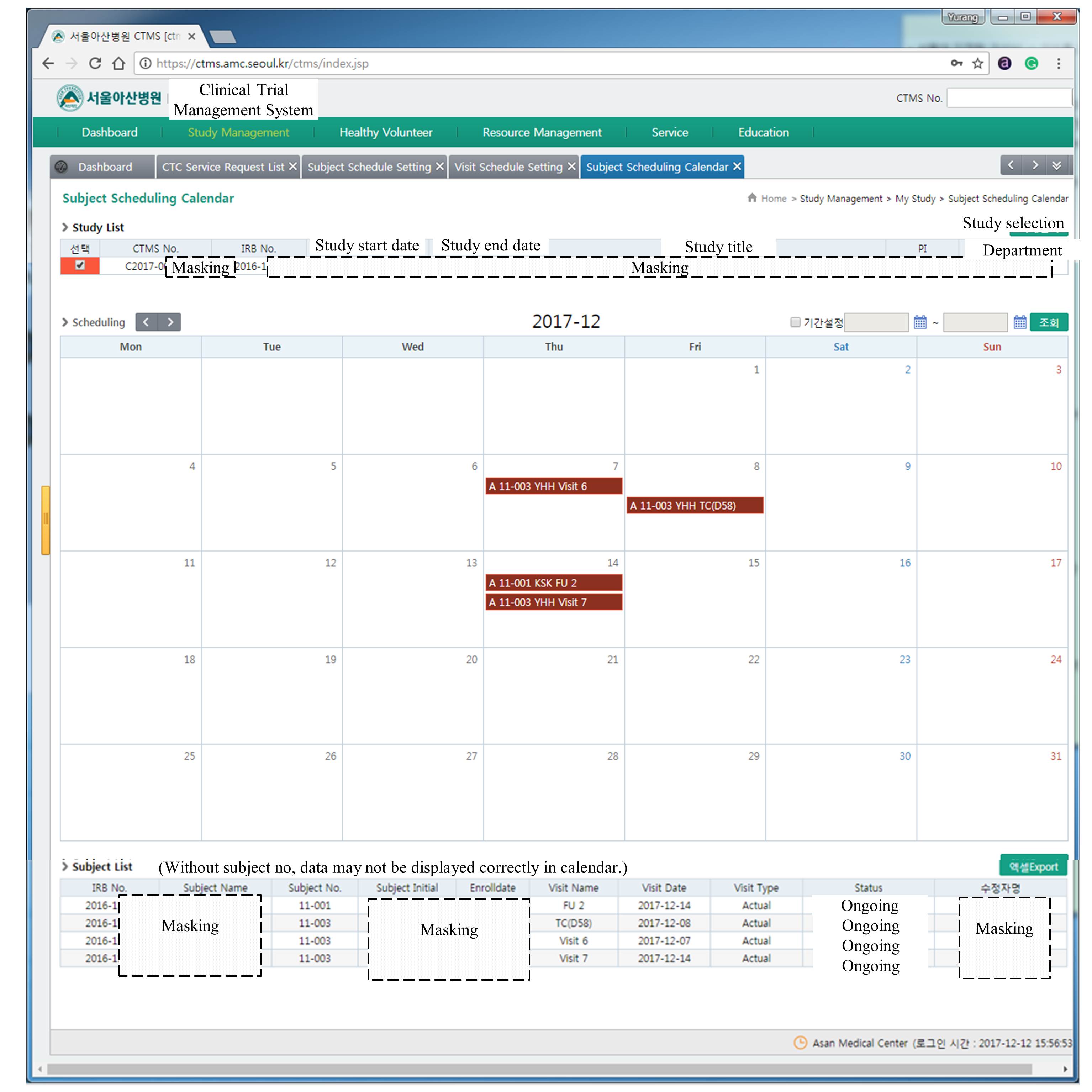

Supplement: Multimedia Appendix 2 [file jmir_v20i4e103_app2.jpg]

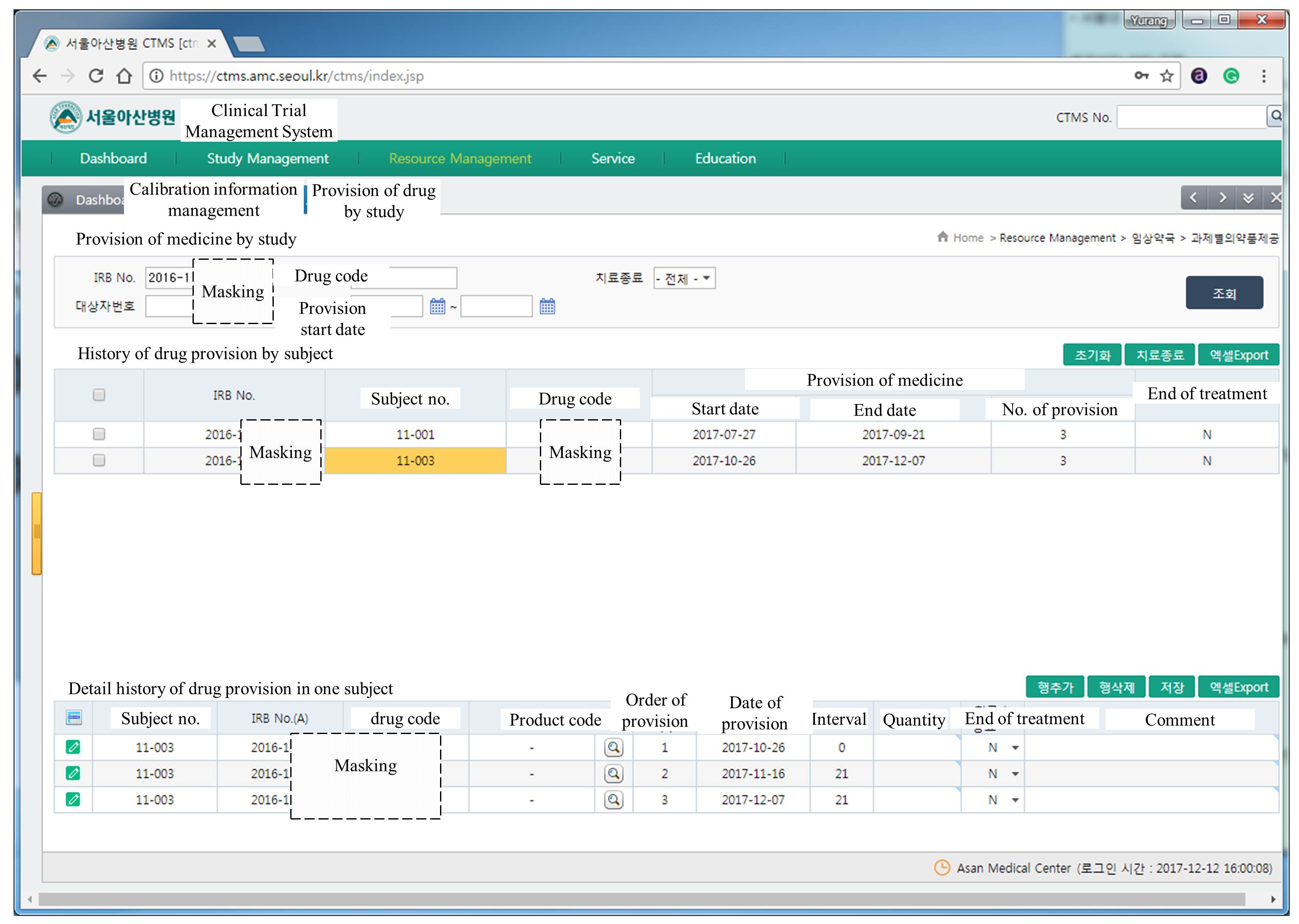

Supplement: Multimedia Appendix 3 [file jmir_v20i4e103_app3.jpg]
